# Effects of AR-Based Home Appliance Agents on User’s Perception and Maintenance Behavior

**DOI:** 10.3390/s23084135

**Published:** 2023-04-20

**Authors:** Takeru Baba, Naoya Isoyama, Hideaki Uchiyama, Nobuchika Sakata, Kiyoshi Kiyokawa

**Affiliations:** 1 Graduate School of Science and Technology, Nara Institute of Science and Technology, 8916-5 Takayamacho, Ikoma 630-0192, Nara, Japan; 2 Faculty of Advanced Science and Technology, Ryukoku University, 1-5 Yokotani, Seta Oe-cho, Otsu 520-2194, Shiga, Japan

**Keywords:** animacy perception, discomfort reduction, mome appliances, intelligence, augmented reality

## Abstract

Maintenance of home appliances can be tedious. Maintenance work can be physically demanding and it is not always easy to know the cause of a malfunctioning appliance. Many users need to motivate themselves to perform maintenance work and consider it ideal for home appliances to be maintenance-free. On the other hand, pets and other living creatures can be taken care of with joy and without much pain, even if they are difficult to take care of. To alleviate the hassle associated with the maintenance of home appliances, we propose an augmented reality (AR) system to superimpose an agent over the home appliance of concern who changes their behavior according to the internal state of the appliance. Taking a refrigerator as an example, we verify whether such AR agent visualization motivates users to perform maintenance work and reduces the associated discomfort. We designed a cartoon-like agent and implemented a prototype system using a HoloLens 2, which can switch between several animations depending on the internal state of the refrigerator. Using the prototype system, a Wizard of Oz user study comparing three conditions was conducted. We compared the proposed method (Animacy condition), an additional behavior method (Intelligence condition), and a text-based method as a baseline for presenting the refrigerator state. In the Intelligence condition, the agent looked at the participants from time to time as if it was aware of them and exhibited help-seeking behavior only when it was considered that they could take a short break. The results show that both the Animacy and Intelligence conditions induced animacy perception and a sense of intimacy. It was also evident that the agent visualization made the participants feel more pleasant. On the other hand, the sense of discomfort was not reduced by the agent visualization and the Intelligence condition did not improve the perceived intelligence or the sense of coercion further compared to the Animacy condition.

## 1. Introduction

People use home appliances to make their daily lives more convenient. In exchange for their usefulness, home appliances require maintenance. For example, when using a refrigerator, it is necessary to fill it in with food items and monitor the expiration dates and condition of the food, and periodically clean the interior. Some people find such maintenance work tedious, and they need extrinsic motivation to do the task. To reduce discomfort when users work with home appliances, we propose a state visualization method that motivates them to do the maintenance work and assists them in deciding what to perform. To this aim, we exploit the effect of animacy perception. Animacy perception is the tendency for observers to perceive inanimate objects as animate. It has effects such as making people feel intimacy for the observed object, empathizing with it, and paying more attention to it [1,2]. To induce animacy perception in the observed object, it is effective if the object has body parts such as limbs and a face [3,4]. In addition, it is effective if the object moves in an unpredictable way or interacts with the surrounding environment [5,6,7]. Therefore, we can expect that animacy perception will be induced in users by adding body parts such as limbs and a face to a home appliance and by making it move according to its internal state. This will make the user feel intimacy for the home appliance and want to take care of it, thus reducing discomfort. We can then indicate the state of home appliances by body movements and facial expressions just like actual living creatures. For example, the arms can scrub where it gets dirty and the face can frown when it is overheated. In our proposed system, we use Augmented Reality (AR) glasses to visualize such features on the home appliance. It is anticipated that AR glasses will become more widespread as they adopt a smaller and more comfortable form factor, enabling many people to wear them for extended periods every day. Based on this assumption, we propose an effective application for potential future use and verify its effectiveness through a user study.

In this research, we developed a new AR state visualization system that superimposes agents of home appliances. We investigated whether the appearance and behavior of the agent reduce the discomfort and the sense of coercion felt by the user. The major contributions of this research are as follows:

- The novel concept of home appliance agents assisting users in maintenance actions through their behavior;
- The development of an AR system that superimposes home appliance agents to make real home appliances appear as living creatures;
- Elucidation of a trend that users will feel intimacy and pleasure when taking care of their home appliances through home appliance agents.

## 2. Related Work

### 2.1. AR-Based User Assistance for IoT Devices

Our aim is to add a highly expressive visualization feature to home appliances because most home appliances do not have any display that can show the internal state. AR glasses can be used to display information about home appliances, even for those that lack a display. GuideMe [8] by Müller et al. and HoloHome [9] by Mahroo et al. are systems that take advantage of AR glasses. They enable users to grasp sensing information related to the location of the installed IoT devices through the AR glasses. Bittner et al. proposed a system that uses AR, LEDs, and handheld devices to present soil humidity information to assist in watering plants [10]. The results of a user interface (UI) evaluation using a questionnaire called AttrakDiff [11] (http://attrakdiff.de/sience-en.html, accessed 19 March 2023) showed that the practical quality of the LED was higher than that of the handheld AR due to the complexity of the operation. However, AR glasses were preferred for their practicality in using both hands for watering and for their portability when changing the viewpoint. Inomata et al. proposed a system that allows users to check the state of home appliances using AR and remotely control them using gestures [12]. As a result of a questionnaire survey on 3D models that are convenient for operation, they found that “the method that uses the actual shape of the product” and “the method that uses motion characters with the same characteristics as the product” were preferred. The results showed that there was no increase in stress due to such state visualization. Participants preferred the condition that placed the 3D model close to the actual home appliance. Bonnani et al. proposed a projection-based interface as an example of a direct AR display on home appliances [13].

### 2.2. Information Visualization in AR

The effects of AR information presentation to assist user actions will depend on the visualization method and content. Wang et al. investigated the relationship between sound and agent shape for AR information visualization. A small human agent was found to be the most preferred when participants were being assisted with tasks [14]. Human-shaped agents easily attracted participants’ attention. However, they were also salient for their uncanny appearance. AR agents who exhibit eye contact were often preferred; however, it should be noted that eye contact could also distract the user’s concentration [15]. Yoshii et al. investigated the persuasiveness of superimposing human-shaped agents near objects by changing their appearance and position [16]. The results showed that the condition in which an agent with the features of the object of concern was placed in the object’s immediate vicinity was most likely to promote a user action. Kim et al. showed that the body gesture and locomotion behavior of an AR agent exhibiting awareness of the surrounding real world improves the perceived social presence of and confidence in the agent [17].

It is difficult for appearance-constrained robots such as aerial robots to convey intent through social cues such as gestures and gaze behaviors. Walker et al. propose a series of explicit and implicit designs that use AR to visually indicate the robot’s motion intentions [18]. Through user evaluation, they found that some AR designs significantly improved objective task efficiency over baselines that receive only physically embodied directional cues. Katzakis et al. propose using a robotic puppet agent to present information about movement through body cues and paths [19]. They experimented in Virtual Reality while assuming that it would be displayed on AR glasses. As a result, they found that the proposed method has advantages. As shown above, even robots that lack expressive capabilities can be augmented by using AR.

### 2.3. Human-Dependent Robots and Intentional Stance

We empirically consider that the higher intimacy for an object, the less unpleasant the maintenance actions related to that object will be. Hence, high intimacy is required to reduce discomfort. Osawa et al. proposed a direct anthropomorphism method that attaches a few liquid crystal displays (LCDs) to the object itself to show eyes and arms, instead of presenting information through a robotic agent [2]. In an experiment in which participants learned object features, the direct anthropomorphism condition increased participants’ attention to the object and improved intimacy compared to the condition in which explanations were given through a robotic agent. Chen et al. proposed a robot agent called Nukabot to remind users of regular maintenance of pickles [20]. Such robot agents can induce a strong intimacy by adding creature-like features directly to the object of concern. Khaoula et al. proposed a “human-dependent” robot to induce such intimacy and to complete a complex task by exhibiting help-seeking behaviors [21]. For example, a garbage can robot created by Yamaji et al. has a weakness that prevents it from achieving its goal of picking up garbage by itself, but it can achieve its goal by drawing assistance from people nearby [22]. There is evidence that the Roomba vacuum cleaner also fosters the user’s cleaning behavior by developing the user’s intimacy for it [23]. Such intimacy helps users deal with Roomba’s poor mobility performance. Here, users voluntarily decide to help the robot, feel the desire to act, and are pleased with the joy of helping it. However, when an actuator is attached to a device as in the studies above, several problems may be introduced such as the noise of motors, an increase in the device and maintenance cost, and awkward and unnatural movements.

To solve these problems, we introduce AR-based home appliance agents overlaid on the actual home appliances. Similar to human-dependent robots, our proposed system attempts to create an intimate relationship between home appliances and the users. Depending on the degree of their weakness, we believe that those around them will feel the motivation to act. Dennett suggested the presence of an intentional stance which is the object’s attempt to act with intention [24]. An intentional stance occurs when the object appears to have an intention or interacts with the environment. Fukuda et al. showed that an intentional stance is generated by random behavior that follows 1/f fluctuations [7]. Matsushita et al. showed that a weak robot, called Talking-Ally, can generate an intentional stance by turning its face to look at humans and attempting to interact with them [25]. It has also been shown that turning one’s face and eyes toward a person with moderate frequency can increase friendliness and attractiveness [26,27]. Therefore, it is possible that the agent’s occasional observation of the user’s condition may generate an intentional stance and further motivate the user to help the agent.

## 3. Proposed System

### 3.1. Usage Scenarios

We propose a method to present the internal state of home appliances by adding characteristics that generate animacy perception to motivate the user to take care of them and help make decisions about what actions to perform. The proposed system is intended to be used by people who feel annoyed with maintaining home appliances or do not feel satisfied after maintaining them. By reducing the discomfort that users have with repetitive household chores, the system will help the users maintain them carefully and periodically while reducing the amount of work that needs to be done at once.

Let us take a refrigerator and a vacuum cleaner as example usage scenarios of the proposed method. A refrigerator agent may complain of pain while holding its position when there is expired food, or express anger when the door is left open. The refrigerator agent will express joy when the user solves the problem and go to sleep with a relieved expression. The facial expressions of a vacuum cleaner agent may change according to its suction power and the amount of vacuumed objects. The user can learn how to vacuum effectively from these expressions and enjoy cleaning together with the vacuum agent.

### 3.2. System Requirements and Approaches

We believe that our proposed system should satisfy the following four requirements in order to reduce discomfort when using home appliances.

I. It can be used with any existing home appliances;
II. It induces the user’s intimacy for the home appliance and motivates them to act on it;
III. It enables the user to easily guess the condition to be dealt with;
IV. It does not give the user a sense of coercion so that the timing of the act can be adjusted comfortably.

In order to satisfy the above four requirements, the proposed system takes the following solutions. To satisfy requirement I, we use AR that can superimpose information without modifying the home appliances themselves. To satisfy requirement II, we add the animacy-inducing appearance and behavior of an agent to the home appliances. To satisfy requirement III, we visualize the home appliance state by corresponding help-seeking body movements and facial expressions. To satisfy requirement IV, we make the agent aware of the user’s busyness and adjust the timing of interruptions.

### 3.3. System Architecture

**Figure 1:**
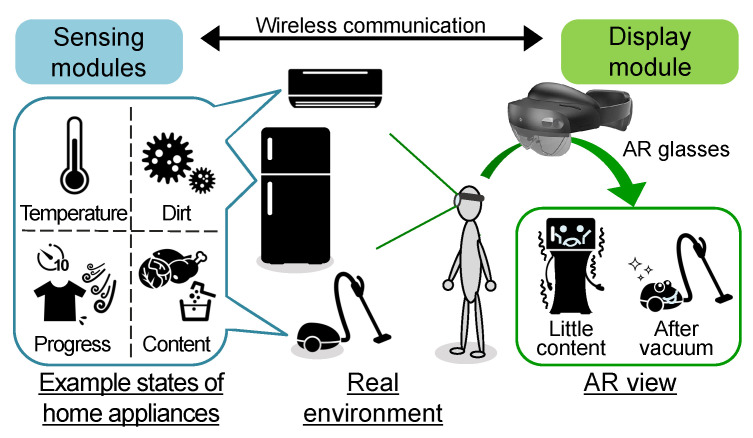
System architecture.

Figure 1 shows a schematic system architecture of the proposed method.

The system consists of sensing modules which acquire the state of the home appliances and a display module to visualize the state through AR glasses. We can utilize sensors embedded in the home appliances if available or retrofit a sensing module to obtain information that is not available through the home appliances themselves. These sensing modules periodically acquire information such as temperature, cleanliness, and remaining consumables and send it to the display module through wireless communication such as WiFi or Bluetooth. Note that the system architecture here is simple and abstract. In reality, a sensor module may consist of multiple sensor devices and a computer that infers a high-level state from multiple low inputs. We are primarily interested in the user’s response to the home appliance agents under a hypothetical situation where the state recognition is accurate. The actual implementation of sensing modules is out of the scope of this research.

In the display module, the SLAM function of the AR glasses is used to recognize the location of the home appliances. Then, the home appliance agent is superimposed on the actual home appliance. The agent’s 3D model has limbs, a face, and additional features added to the target home appliance. Examples of the additional features include body deformation, a tail, and sound effects such as voice. The agent is animated so that it appears as a living creature to visualize the appliance’s state.

We used Unity 3D to develop the prototype system and a HoloLens 2 (field of view: 43×29 [deg], resolution: 1440×936 [px]) to visualize the AR state visualization. We used VisionLib (https://visionlib.com, accessed 19 March 2023) to recognize the location of a 3D marker attached to the home appliance. We created a 3D model of the home appliance agent with Blender according to the discussions in Section 4 and the actual home appliance (refrigerator) used.

## 4. Design of the Refrigerator Agent

### 4.1. Overview of the Focus Group

The appearance and behavior of the refrigerator agent should be determined to feel natural to most people. Unnatural appearance and behavior can hinder a user’s feeling of intimacy for their refrigerator. Therefore, in this research, we used focus groups where several people discussed and came up with ideas for the agent design and summarized their opinions. The participants discussed the question “what would a refrigerator look like if it was alive?” The discussion was held online with a whiteboard function to draw the appearance of the refrigerator agent. The participants were 17 males and 2 females between 22 and 28 years of age.

In order to incorporate a large number of opinions, the participants were divided into groups. To consolidate the opinions of each group into one proposal, a tournament system was used. First, four groups of four or five people each discussed their ideas, then representatives from each group discussed them in one group to finalize the ideas for the appearance and behavior of the refrigerator agent.

### 4.2. Results of Final Focus Group

**Figure 2:**
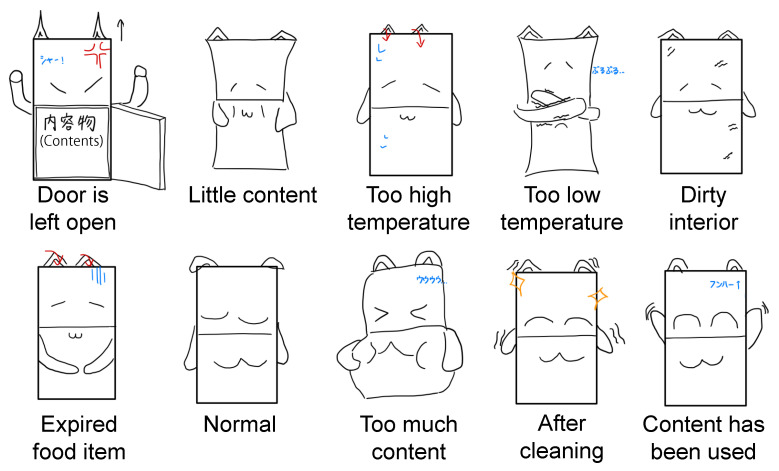
The design of the refrigerator agent discussed in the final group discussion.

A tracing of the images of the refrigerator agent that resulted from the second-round discussion is shown in Figure 2.

The elements to be added were body deformation, limbs, a face, and a voice. In the discussion, arms were added first as “gestures can improve expressiveness”. In addition, ears were added for the same reason of “increasing expressiveness”. Then the face was added because “it is unnatural to have arms but no face”. The body deformation was added as “it is a universal function of a living thing and it can be expressed naturally”. In addition, the voice was added as a sub-element, “so that important information could be conveyed to the environment”. Throughout the discussion, there was a consensus that “a human-like appearance would be creepy”, so they wanted to abstract the elements to be added. Thus, the arms were round and simple in shape, and the facial expressions were represented as emoticons.

## 5. Experiment

### 5.1. Overview

We hypothesized that generating animacy perception in a home appliance will reduce the user’s discomfort. In order to further reduce discomfort, it is preferable to be able to start working on the home appliance at a time desired by the user. Furthermore, it is not desirable for the problem to be ignored for a long time. It is thus important to have an appropriate guiding effect that makes the user want to start the maintenance work at an appropriate time without feeling a sense of coercion. Examples of appropriate timing include when a user finishes reading a book chapter or stops studying due to a difficult problem.

Therefore, we wanted to investigate the effectiveness of not only the creature-like agent’s behavior itself but also the agent’s awareness of the user’s busyness. To investigate these two factors, we compared two types of agent conditions with the text-only condition. In the *Animacy* condition, the agent’s behavior changes solely based on the internal state of the home appliance, completely ignoring the user’s situation. Specifically, the face of the refrigerator agent is always fixed in the frontal direction and does not pay attention to the surrounding environment. On the other hand, in the *Intelligence* condition, the agent’s behavior changes not only based on the internal state of the refrigerator but also on the user’s busyness. The agent’s behavior is made to appear curious about the user’s situation. The agent looks at the user sometimes and the level of the agent’s friendliness to the user is visualized as a heart icon near the agent. We hypothesized that the Intelligence condition would further reduce the user’s discomfort and it could also reduce the sense of coercion that the user in the Animacy condition would feel.

In the experiment, we investigate the following five research questions (RQs).

RQ1: Does the proposed system induce animacy perception and a sense of intelligence toward the agent?
RQ2: Can the user easily guess the state of the home appliance?
RQ3: How do home appliances’ appearance and behavior influence users’ discomfort?
RQ4: Does intelligent behavior reduce the sense of coercion?
RQ5: Which condition increases the frequency of users’ maintenance?

### 5.2. Task Design

The experiment was conducted as a between-subject design in order to avoid order effects. In the experimental task, AR visualizations were displayed according to the condition of the refrigerator, and the participants actually performed the maintenance operations when they wanted to act. We employed the Wizard of Oz (WoZ) method to investigate the effectiveness of AR visualizations. This was to avoid the influence of imperfect state recognition and to present different states in a short period of time to the participants in a consistent and controlled manner. That is, the experimenter changed the refrigerator state and corresponding AR visualizations without using actual sensing modules.

In determining the details of the experimental task, the following three requirements were established.

I. AR visualizations should be in the participants’ view while they are working on a different job.
II. Each state and the corresponding AR visualization should be presented at least once at a time when the participants can take a short break.
III. The maintenance task of the refrigerator should be easy to guess from the AR visualizations and easy to accomplish.

In order to satisfy requirement I, participants were asked to perform a Visual Display Terminals task (VDT task) in parallel with the refrigerator maintenance task so that they could see the refrigerator right next to the desktop monitor for the VDT task. By having the participants transcribe the sentences presented on the left side of the monitor to its right side, they were made to frequently move their gaze horizontally, so that the AR visualizations of the refrigerator became easily visible even in the narrow field of view of the HoloLens 2. For requirement II, we used a transcription task of a novel that spanned many pages to allow the participants to work for an extended period of time. Assuming that they would take short breaks as they turn the pages, the number of words transcribed by each participant was adjusted so that such a short break would occur at least once per refrigerator state. To fulfill requirement III, we placed corresponding items around the refrigerator. These items included a rag for cleaning the refrigerator, a trash can for disposing of expired food items, and a shopping bag containing the food items to be replenished in the refrigerator.

### 5.3. Experimental Settings

#### 5.3.1. Refrigerator States

**Figure 3:**
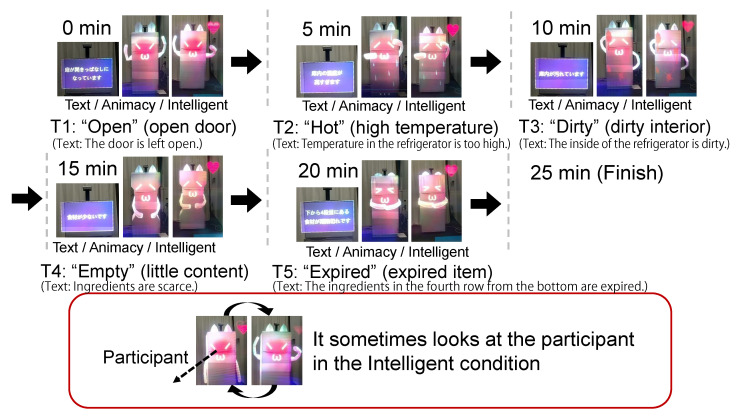
AR state visualization in each subtask (T1–T5). (**Left** in the triplet) Text condition, (**middle** in the triplet) Animacy condition, (**right** in the triplet) Intelligence condition.

We selected three regular states (*Normal*, *Full*, *Joy*) and five irregular states (*Open*, *Empty*, *Hot*, *Dirty*, *Expired*) through the focus group discussion in Section 4. Figure 3 illustrates the AR visualizations corresponding to the five irregular states. *Normal* is a state in which there is no problem. *Full* is a state in which there is much content. *Joy* is a state in which it has recovered from an irregular state. *Open* is a state in which the door is left open. *Empty* is a state in which there is little content. *Hot* is a state in which the internal temperature is too high. *Dirty* is a state in which the interior is dirty. *Expired* is a state in which it contains an expired food item. In the *Dirty* and *Expired* states, the agent’s arms scrub around the corresponding position.

#### 5.3.2. Intelligent Behavior

In the Intelligence condition, the agent sometimes turned its face to the participant. The timing of the head turn was determined based on the 1/f fluctuation to facilitate animacy perception. To derive the 1/f fluctuation, we used the intermittent chaos as shown in Equation (1) [28].

(1) $$x=\left\{\begin{array}{cc} x+2x^{2} & \left(0\leq x\leq 0.5\right)\\ x-2{\left(1-x\right)}^{2} & \left(0.5<x\leq 1\right) \end{array}\right.$$

where *x* fluctuates in the closed interval [0, 1] and a threshold of 0.5 was used to determine whether or not to face the participant. As the fluctuation becomes too small when *x* approaches either end of the interval, *x* was reset to a random value in the range [0.1, 0.2] and [0.8, 0.9] when it became smaller than 0.1 or larger than 0.9, respectively. In our implementation, *x* was updated at 100 Hz, synchronized with the rendering process.

In the Intelligence condition, a heart icon was shown at the upper right corner of the refrigerator to visualize the refrigerator’s affection for the participant. The heart icon indicated the level of affection as a red vertical meter. It was 0% in the beginning, went up by 10% every time the participant solved a task, and went down by 25% every time the refrigerator was in an irregular state.

#### 5.3.3. Maintenance Task

Figure 3 illustrates five subtasks (T1–T5) for the refrigerator maintenance and the corresponding AR state visualization styles in three conditions—Text, Animacy, and Intelligence.

AR visualizations corresponding to the irregular states in each of the subtasks were presented only for three minutes, and it went back to the normal state if three minutes had elapsed or the participants addressed the problem properly.

The joy state was presented for 20 s when the participant accomplished a subtask such as closing the door, then the state transitioned back to the normal one.

#### 5.3.4. Transcription Task

**Figure 4:**
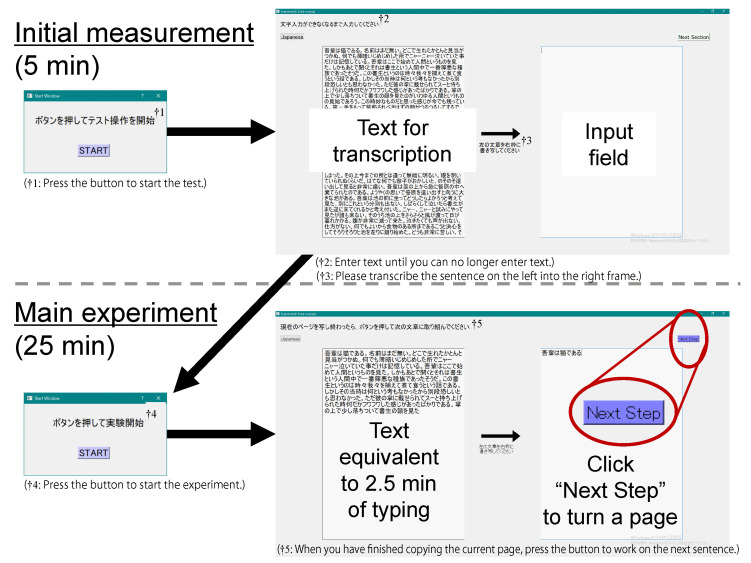
Transcription interface.

As we wrote in Section 5.2, the participants were asked to perform a transcription task of a novel (“I am a Cat” by Soseki Natsume (https://www.aozora.gr.jp/cards/000148/files/789_14547.html, accessed 19 March 2023)) spanning many pages shown on a desktop monitor in parallel with the refrigerator maintenance task. The novel was selected for its plain writing and simple kanji characters used. They transcribed the text presented on the left side of the monitor to its right side. The number of words transcribed by each participant was adjusted so that a page turn would occur at least once per instance of the irregular states (T1–T5). In the first five minutes of the experimental task, no problem occurred in the refrigerator and the typing speed of the participant was measured by the transcription interface shown in Figure 4 (top). In the subsequent transcription task that lasted for 25 min, text equivalent to 2.5 min of typing was shown per page based on the initial typing speed (see Figure 4 (bottom right)). The participants needed to click the “Next Step” button to proceed to the next page every time they finished transcribing the displayed text. The “Next Step” button appeared only after more than 90% of the text had been entered to avoid double clicking. While we instructed them to transcribe the text as accurately as possible, no error was checked and they could proceed with the task with imperfect transcription.

#### 5.3.5. Layout

**Figure 5:**
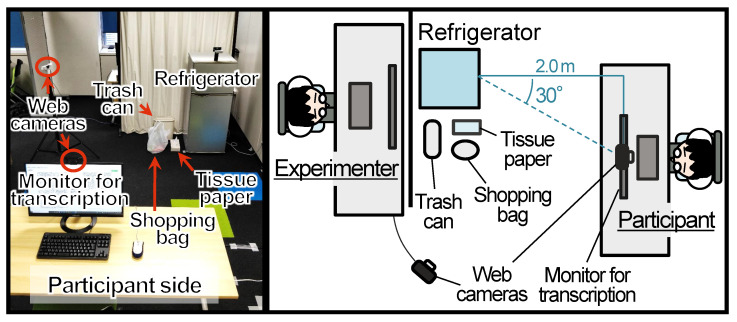
Layout of the experiment.

The experiment was conducted in the environment shown in Figure 5.

In order to accomplish the maintenance task, a few items (a trash can, tissue paper, and a shopping bag) were placed on the left side of the refrigerator. The refrigerator (SHARP SJ-H12Y, W480 × D480 × H1150 mm) was positioned 2.0 m behind and 30 deg to the right of the desktop monitor for the transcription task. The experimenter sat behind the refrigerator and a partition so that they were not visible to the participants. They watched the participants through a web camera and changed the refrigerator states to conduct the WoZ experiment.

The actual food and beverage items were placed inside the refrigerator and had stickers attached indicating their expiration dates. The right side of the refrigerator was set at a higher temperature. The participants could lower the temperature by adjusting the dial.

Another partition was placed in front of the refrigerator and other items until the beginning of the experiment so that participants could not guess the problems. Before each participant started the experiment, the temperature was set to high, the refrigerator interior was dripped with colored water indicating dirt, an expired food item was placed on the lower side of the refrigerator, and the door was left open a little.

#### 5.3.6. Procedure

Before the experiment started, the experimenter confirmed that the HoloLens 2 was tracking the refrigerator with decent registration accuracy and that the AR visualization could be changed through the the server PC. Then each participant was asked to sit on the chair, fill in a consent form, put on the HoloLens 2 and a heart rate monitor (Polar Verity Sense (POLAR) wristwatch), and do the calibration for gaze tracking. They were then given instruction for the maintenance and transcription tasks and their typing speed and heart rate variability (HRV) were measured for five minutes to set their baseline values. The instruction included that the transcription task needs to be done page by page, that they are allowed to stand up and move to the refrigerator and use the provided items to complete the maintenance task, and that it is optional to act or not. Immediately before the experiment, they were shown a printed table summarizing the correspondence between the refrigerator states and AR visualizations and asked to memorize them for about one minute. The experimenter then moved the partition in front of the refrigerator, rang the start bell, and the participant started the experiment by clicking the start button (Figure 4 (bottom left)). At the end of the 25-min long experiment, they were asked to answer the questionnaire.

#### 5.3.7. Metrics

For each participant, head position, gaze position on the refrigerator, HRV and typed text and its timing were recorded as well as the questionnaire results. The LF/HF ratio was calculated from the HRV as an indicator of the stress level.

**Figure 6:**
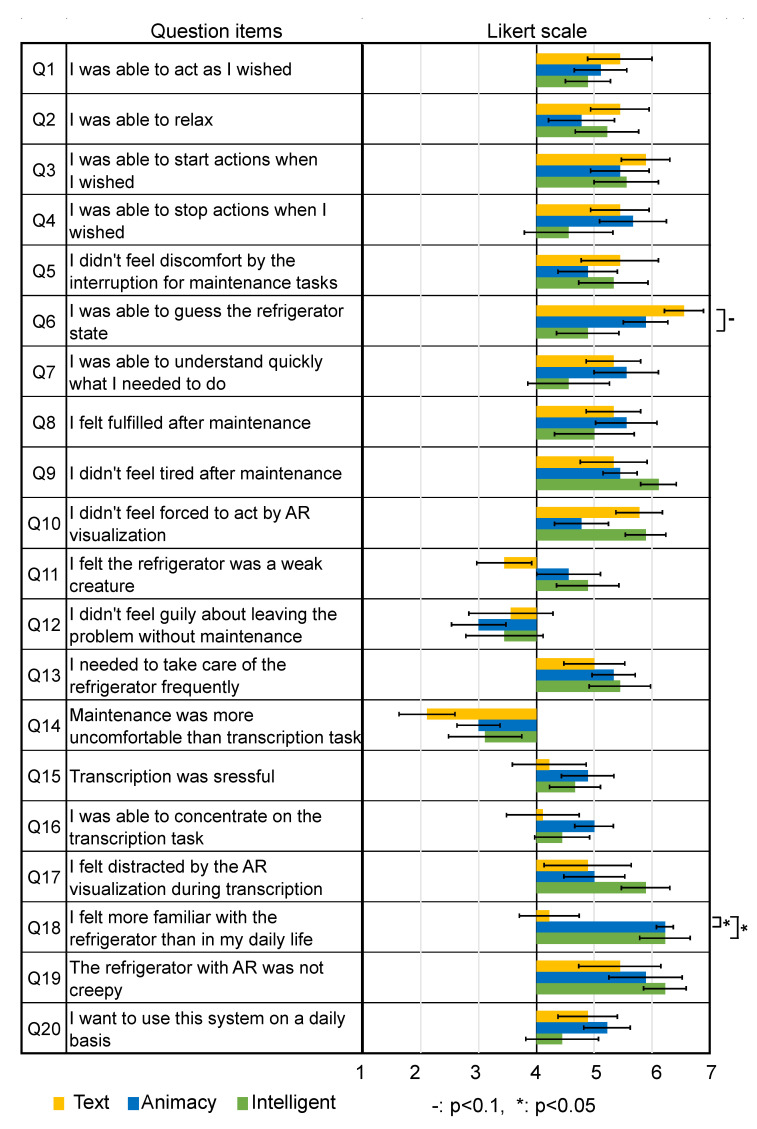
Results of original question items.

The questionnaire consisted of AttrakDiff [11], 24 items from Godspeed Questionnaire [29], and 20 original questions. AttrakDiff contained 28 items on a 7-point SD (Semantic Differential) scale index in four categories (practical quality (PQ), hedonic quality identification (HQ-I), hedonic quality stimulation (HQ-S), and attractiveness (ATT)). The Godspeed questionnaire contained 24 items on a 5-point SD scale index in five categories (anthropomorphism, animacy, likeability, perceived intelligence, and perceived safety). Our 20 original questions were on a 7-point Likert scale summarized in Figure 6.

**Figure 7:**
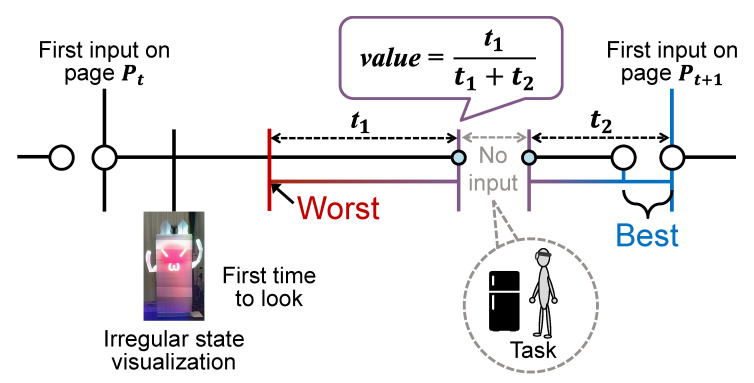
Evaluation of action timing.

We evaluated the action timing of the maintenance task as an indicator of the sense of coercion. Our hypothesis was that the participants would start the maintenance task at the time of a page turn if they were feeling little pressure and they were focusing well on the transcription task, and that they would start the maintenance task as soon as they noticed the AR visualization if they were feeling much pressure and they were not focusing on the transcription task. Thus, we calculated the action timing value for each irregular state by Equation (2) from the time interval t1 (from the first gaze at the refrigerator to the start of the leaving the seat) and the time interval t2 (from the end of leaving the seat to the end of the transcription task on that page) as shown in Figure 7. Our assumption was that the higher the sense of coercion, the lower this value would be.

(2) $$\begin{array}{cc} value= & \frac{t1}{t1+t2} \end{array}$$

#### 5.3.8. Participants

The experiment was approved by the ethical review committee of our institution. Since the experimental task involved a transcription task of a Japanese novel, only those who could read and type Japanese were recruited through on-campus announcements. In total, 31 participants (24 males and 7 females aged between 21 and 30) took part in the experiment. We excluded the data of four participants whose registration accuracy was found to be too low or who ignored the AR visualization for more than 10 min from the start of the experiment. In the end, we analyzed the results of nine participants for each of the three conditions Text, Animacy and Intelligence.

### 5.4. Results

#### 5.4.1. Objective Evaluation

**Figure 8:**
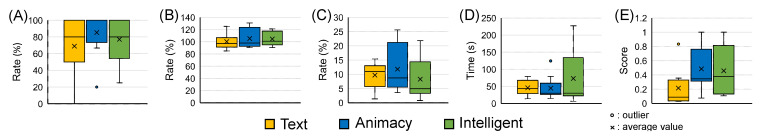
Results of objective evaluation. (**A**) Subtask success rate (%). (**B**) Relative change of the LF/HF ratio (%). (**C**) Percentage of time spent looking at the refrigerator while seated (%). (**D**) Time interval from state visualization to leaving the seat (s). (**E**) Action timing values.

As our emphasis is on how the participants respond to the state visualization, it was considered an unsuccess if they misunderstood what to do on the state visualization or solved a different problem other than what was being visualized. In such a case, the recovery state (Joy) was not presented. Figure 8A shows the success rate for each condition. In the Text condition, we observed that many participants ignored the state visualizations and did not act on them. In the Intelligence condition, we observed that many participants looked at the state visualizations and acted on them, but gave up or solved another problem because they did not understand what they needed to do. Figure 8B shows the relative change of the LF/HF ratio from the baseline to that during the main transcription task for each condition. Regardless of the condition, it was higher than 100% for most of the participants, suggesting a higher stress level. Figure 8C shows the percentage of time in which the participants were looking at the refrigerator while seated for each condition. Figure 8D shows the time interval between the beginning of each irregular state visualization to the start of the leaving the seat for each condition. Figure 8E shows the action timing values calculated by Equation (2). Welch’s *t*-test and the Benjamini–Hochberg (BH) correction method did not find any statistically significant differences in any condition pair in any of these five evaluation metrics.

#### 5.4.2. Subjective Evaluation

**Figure 9:**
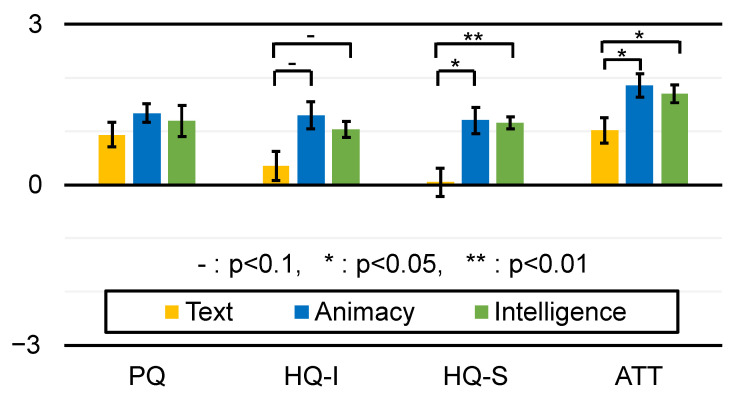
Results of AttrakDiff in each category.

**Figure 10:**
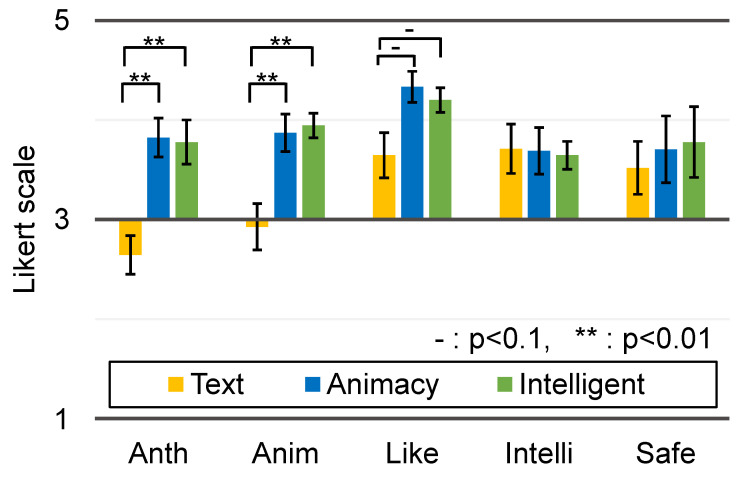
Results of the Godspeed Questionnaire in each category.

Figure 9 summarizes the results of AttrakDiff in each category, Figure 10 summarizes the results of the Godspeed Questionnaire in each category, and Figure 6 shows the results of the original 20 questions. In these figures, the ratings are considered interval scales for simplicity. The error bars indicate the standard error.

Regarding the results of AttrakDiff, Wilcoxon’s rank sum test and the BH correction method found statistically significant differences between the Text and Animacy conditions and between the Text and Intelligence conditions in the HQ-S category. For the HQ-S category, the Animacy condition was considered more stimulating and pleasant than the Text condition (n=15,z=2.28,p<0.05) and the Intelligence condition was considered more stimulating and pleasant than the Text condition (n=6,z=3.23,p<0.01). For the ATT category, the Animacy condition was considered more attractive than the Text condition (n=16,z=2.2168,p<0.05) and the Intelligence condition was considered more attractive than the Text condition (n=15,z=2.23,p<0.05). Wilcoxon’s rank sum test and the BH correction method also found trends toward significance between Text and Animacy conditions (n=18,z=1.98,p<0.1) and between the Text and Intelligence conditions (n=14,z=2.38,p<0.1) in the HQ-I category. No statistically significant differences were found between the Animacy and Intelligence conditions in any of the categories.

Regarding the results of the Godspeed Questionnaire, Wilcoxon’s rank sum test and the BH correction method found statistically significant differences between the Text and Animacy conditions and between the Text and Intelligence conditions in the Anthropomorphism and Animacy categories. For the Anthropomorphism category, the Animacy condition was considered more human-like than the Text condition (n=2.5,z=3.77,p<0.001) and the Intelligence condition was considered more human-like than the Text condition (n=6,z=3.18,p<0.01). For the Animacy category, the Animacy condition was considered more creature-like than the Text condition (n=12,z=2.58,p<0.01) and the Intelligence condition was considered more creature-like than the Text condition (n=10,z=2.83,p<0.01). For the Likeability category, the Animacy condition was considered more likable than the Text condition (n=15,z=2.26,p<0.05) and the Intelligence condition was considered more likable than the Text condition (n=18,z=2.00,p<0.05). No statistically significant differences were found between the Animacy and Intelligence conditions in any of the categories.

Regarding the original 20 questions, Wilcoxon’s rank sum test and the BH correction method found statistically significant differences between the Text and Animacy conditions (n=11.5,z=2.84,p<0.05) and between the Text and Intelligence conditions (n=14,z=2.37,p<0.05) only in Q18 (“I felt more familiar with the refrigerator than in my daily life.”). Wilcoxon’s rank sum test and the BH correction method also found a trend toward significance (n=65.5,z=2.39,p<0.1) between the Text and Intelligence conditions in Q6 (“I was able to guess the refrigerator state.”). No statistically significant differences were found between the Animacy and Intelligence conditions in any questions.

### 5.5. Discussion

#### 5.5.1. RQ1: Does the Proposed System Induce Animacy Perception and Sense of Intelligence toward the Agent?

The results of the Anthropomorphism and the Animacy categories of the Godspeed Questionnaire (Figure 10) as well as the results of the original question item Q18 (Figure 6) clearly show that the home appliance agent induces animacy perception. On the other hand, there were no statistically significant differences between the Animacy and Intelligence conditions, suggesting that a higher sense of intelligence was not induced by the Intelligence condition compared to the Animacy condition.

#### 5.5.2. RQ2: Can the User Easily Guess the State of the Home Appliance?

The PQ (practical quality) of AttrakDiff in the Animacy and Intelligence conditions was not inferior to that in the Text condition, even though the participants checked the correspondence table regarding the AR visualization styles and the refrigerator states for only about one minute.

However, the rating of Q6 (“I was able to guess the refrigerator state.”) tended to be lower in the Intelligence condition compared to that in the Text condition (n=65.5,z=2.39,p<0.1). Indeed, we observed that many participants were unable to understand the refrigerator state that was visualized in Intelligence condition. We speculate that this is partly due to the fact that the agent in the Intelligence condition lowered its arms without any movement when it was looking at the participants. The agent was looking at the participant for half of the entire time based on the 1/f fluctuation in the Intelligence condition. So, this problem will be alleviated by taking a longer time to remember the agent’s behavior and by reducing the frequency of the looking-back motion.

#### 5.5.3. RQ3: How Do Home Appliance’s Appearance and Behavior Influence User’s Discomfort?

The results of the LF/HF ratio (Figure 8B) show that the Animacy and Intelligence conditions did not reduce the stress level compared to the Text condition. The same can be said from the fact that no statistically significant differences were found in the question items Q1, Q2, Q5, Q8, Q9, Q12, Q15 and Q19 that are related to discomfort reduction. Our hypothesis that the Intelligence condition would (further) reduce the discomfort compared to the Animacy condition was not supported either. On the other hand, some results such as HQ-S and ATT of AttrakDiff clearly show that the agent visualization improved the perceived pleasure and attractiveness compared to the Text condition. That is, adding animacy to a home appliance can make people want to use it more.

#### 5.5.4. RQ4: Does Intelligent Behavior Reduce the Sense of Coercion?

We found no significant differences between Animacy and Intelligence conditions in any of the experimental results, and we cannot say that the Intelligence condition reduces the sense of coercion compared to the Animacy condition.

#### 5.5.5. RQ5: Which Condition Increases the Frequency of Users’ Maintenance?

We found no significant differences among the three conditions in terms of the subtask success rate (Figure 8A), so there was no clear winner in increasing the frequency of user maintenance.

## 6. Discussion

### 6.1. Summary of Findings and Potential Users

From the experimental results, it was found that creature-like appearance and behavior made the home appliances more attractive and the participants feel intimacy for them. It was also evident that the agent visualization made the participants feel more pleasant. On the other hand, we did not find any negative impacts of the agent visualization on the objective and subjective metrics such as the task success rate and the sense of coercion. These findings suggest that the proposed system can potentially be integrated into our daily lives with only positive influences.

As examples of those who would benefit from the proposed system, children can learn how to do housework in a fun manner and those who are unmotivated or bored with housework can be motivated to do the maintenance work. They will acquire the mindset that housework should be done carefully and regularly. We also believe that it could be applied to self-management for people who need to improve their lifestyle. As in Perusquía-Hernández et al.’s work on robot mirroring [30], it is possible for a robot to function as a self-tracking agent that mirrors one’s health status to provide indirect support for their life.

### 6.2. Limitations

The biggest limitation of this research is that this experiment was conducted using the Wizard of Oz (WoZ) method, which does not automatically recognize the actual state of the home appliances or the user. We have begun to build an automatic recognition system for the state of the door, the temperature in the cabinet and the type of food in the cabinet, but it is difficult to recognize all of the conceivable conditions with high accuracy. Our model to infer the appropriate maintenance timing (Equation (2)) is very simple. It is also difficult to precisely estimate the user’s busyness and general psychological states in practical situations.

As another limitation, the refrigerator was placed in front of the participants and there was nothing around it. In reality, home appliances are not always in the user’s view and there are normally other objects around them. Thus, the effectiveness of the AR state visualization will be lower in a more realistic setting.

Although the agent was carefully designed, only one set of appearances and behaviors of the agent for one type of home appliance was studied. Various characteristics of the agent, including appearance, behavior, body parts and voice, need to be studied in detail.

In the experiment, we only examined the short-term effects of using the system for one day. In the long run, we expect that the participants will become bored with the Text condition quickly due to its simple appearance and they will keep the motivation for maintaining the agent conditions thanks to their affection. Such long-term effects need to be investigated in the future.

In the present experiment, there was an unequal representation of male and female participants. The effectiveness of the proposed method may vary depending on gender and prior experience with pet ownership. To better understand the characteristics of the proposed method’s effects, we plan to increase the sample size in future studies. By doing so, we hope to obtain more reliable results that account for potential gender-based differences and variations in pet ownership experience.

## 7. Conclusions

In this paper, to alleviate the hassle associated with the maintenance of home appliances, we proposed an AR system to superimpose a 3D model of the home appliance that makes it look like a living thing, thereby inducing animacy perception in the user. Taking a refrigerator as an example, we verified whether the superimposed display of creature-like behavior motivates the users to perform maintenance and reduces associated discomfort. First, a cartoon-like design with eyes, ears, a mouth and arms was selected through focus group discussions so that the animacy-inducing appearance and behavior would feel natural.

Based on this design, we implemented a prototype system using a HoloLens 2, which can switch between several animations depending on the internal state of the refrigerator. Using the prototype system, we conducted a Wizard of Oz user study that compared the proposed method (Animacy condition), an additional behavior method (Intelligence condition) and a text-based method for presenting the refrigerator’s state. The Intelligence condition exhibited behavior in which an interrupting animation was played only at a time when users could take a break.

The results showed that the proposed method induced animacy perception and a sense of intimacy, but no effects in reducing discomfort were found. In both the Animacy and Intelligence conditions, users felt the refrigerator to be more attractive and the maintenance task to be more pleasant than in the Text condition.

In the future, we would like to implement a fully functioning system including the state recognition of the home appliance and the user. We would also like to investigate the effects of different design possibilities and long-term effects in a more realistic setting. The focus of the experiments detailed in this paper was to characterize the specific properties of AR agents that convey animacy. Although there are alternative methods that do not require AR glasses, such as sending alert emails, using LED lights attached to refrigerators or providing verbal instructions through a speaker, we did not explore these methods in this study. Instead, our aim was to investigate the potential of AR agents in providing animacy. Nevertheless, we recognize the importance of comparing these methods with AR agents to further establish the advantages of AR agents. Consequently, in future studies, we plan to conduct comparative analyses to better understand the benefits of AR agents over other methods.

## Data Availability

The data presented in this study are available on request from the corresponding author.
